# Everybody's Page

**Published:** 1892-10-08

**Authors:** 


### EVERYBODY'S PAGE.

The state of our streets is a constant complaint, and the spread of consumption through the habit of spitting in public places is one of the dangers which dwellers in cities, above all, are liable to. That the present condition of affairs is not impossible of improvement has been proved in New York, Pittsburg, and other American cities, where some public-spirited ladies have organised societies for bringing the streets into better condition as to cleanliness, and have resolutely set themselves to repress the obnoxious habit, which in America is even worse than with us.

Few reforms can be carried out without injury as well as advantage accruing. A difficult nut to crack has presented itself in connection with the Factory Act newly passed restricting the hours of employment for women in Bombay. In Ahmedabad the women employed in the cotton mills have petitioned for exemption in their case, as the law, if carried out, will prevent their employment, and men or boys, to whom the limitation of hours does not apply, will be taken on as mill-minders in their stead. The number of hours' work permissible is eleven, which seems enough indeed; but either more time must be allowed, or large numbers of these trained hands must fall out of employment without having other means of livelihood to turn to.

